# Phosphorylation of VpCAMTA3 by VpCDPK13 enhances cold tolerance in grapevine

**DOI:** 10.1093/hr/uhag121

**Published:** 2026-07-24

**Authors:** Danni Han, Junke Liu, Shutong Wan, Mi Xun, Ruixin Tang, Qiaoqiao Zhang, Chundong Niu, Yinpeng Xie, Yingqiang Wen, Qingmei Guan

**Affiliations:** State Key Laboratory for Crop Stress Resistance and High-Efficiency Production/Shaanxi Key Laboratory of Apple, College of Horticulture, Northwest A&F University, Yangling 712100, China; State Key Laboratory for Crop Stress Resistance and High-Efficiency Production/Shaanxi Key Laboratory of Apple, College of Horticulture, Northwest A&F University, Yangling 712100, China; State Key Laboratory for Crop Stress Resistance and High-Efficiency Production/Shaanxi Key Laboratory of Apple, College of Horticulture, Northwest A&F University, Yangling 712100, China; Shandong Institute of Pomology, Shandong Academy of Agricultural Sciences, Tai'an, Shandong, 271000, China; State Key Laboratory for Crop Stress Resistance and High-Efficiency Production/Shaanxi Key Laboratory of Apple, College of Horticulture, Northwest A&F University, Yangling 712100, China; State Key Laboratory for Crop Stress Resistance and High-Efficiency Production/Shaanxi Key Laboratory of Apple, College of Horticulture, Northwest A&F University, Yangling 712100, China; State Key Laboratory for Crop Stress Resistance and High-Efficiency Production/Shaanxi Key Laboratory of Apple, College of Horticulture, Northwest A&F University, Yangling 712100, China; State Key Laboratory for Crop Stress Resistance and High-Efficiency Production/Shaanxi Key Laboratory of Apple, College of Horticulture, Northwest A&F University, Yangling 712100, China; State Key Laboratory for Crop Stress Resistance and High-Efficiency Production/Shaanxi Key Laboratory of Apple, College of Horticulture, Northwest A&F University, Yangling 712100, China; State Key Laboratory for Crop Stress Resistance and High-Efficiency Production/Shaanxi Key Laboratory of Apple, College of Horticulture, Northwest A&F University, Yangling 712100, China

## Abstract

Low temperature severely limits grapevine growth and productivity. In this study, VpCDPK13, a calcium-dependent protein kinase, was identified as a cold-inducible positive regulator in *Vitis pseudoreticulata*. Protein–protein interaction assays demonstrate that VpCDPK13 physically interacts with the transcription factor VpCAMTA3. Functional analyses reveal that both VpCAMTA3 and VpCDPK13 positively regulate grapevine cold tolerance. Moreover, *in vitro* kinase assays show that VpCDPK13 phosphorylates VpCAMTA3 at Thr588, Ser834, and Ser1049, and this phosphorylation further enhances the biological function of VpCAMTA3 under cold*.* Collectively, these findings establish the VpCDPK13–VpCAMTA3 signaling module as a key regulatory pathway that promotes grapevine adaption to cold stress.

## Introduction

Low temperature is one of the most detrimental abiotic stresses affecting plant growth, development, and productivity worldwide [1]. Grapevine (*Vitis vinifera* L*.*), a perennial fruit crop of major economic importance, is particularly vulnerable to freezing stress due to its relatively narrow cold tolerance range [2, 3]. Cold stress disrupts both vegetative and reproductive development, reduces bud viability, and compromises yield stability across growing seasons [4, 5]. Elucidating the molecular mechanisms underlying grapevine cold tolerance is therefore essential for breeding climate-resilient cultivars.

To cope with low-temperature stress, grapevine activates a suite of molecular and physiological responses [6–8], including osmolyte accumulation, activation of antioxidant system, and induction of secondary metabolite biosynthesis [9–12]. A rapid transient increase in cytosolic Ca^2+^ concentration functions act as a central second messenger that triggers downstream signaling cascades and reprograms cellular physiology [13, 14]. Among Ca^2+^ sensors, calcium-dependent protein kinases (CDPKs/CPKs) constitute a unique family of Ser/Thr kinases that directly bind Ca^2+^ via their calmodulin-like domains, thereby coupling Ca^2+^ perception to protein phosphorylation without intermediary CaM proteins ([15, 16]). This structural feature enables CDPKs to rapidly transduce Ca^2+^ signals through autophosphorylation and phosphorylation of diverse substrates.

Accumulating evidence highlights pivotal roles for CDPKs in cold responses across plant species. In white clover, several *CDPK* genes are strongly upregulated under chilling conditions [17]. In mustard, *BjCDPK10* and *BjCDPK21* are induced by low temperature and contribute to cold-responsive metabolism ([18]). In fruit crops, *EjCDPK25* in loquat (*Eriobotrya japonica*) enhances freezing tolerance by modulating reactive oxygen species (ROS) level [19]. In grapevine, although the functional diversity of CDPKs remains incompletely characterized, available studies suggest these kinases act as central nodes linking Ca^2+^ signals to transcriptional and metabolic regulation during cold stress [3, 20–22].

Beyond CDPKs, calmodulin-binding transcription activator 3 (CAMTA3) functions as a key transcription regulator in plant cold responses [23, 24]. CAMTA3 activity is regulated by Ca^2+^/CaM binding and by phosphorylation. In *Arabidopsis*, CAMTA3 is directly phosphorylated by MPK3 and MPK6, leading to reduced protein stability and attenuation of cold-responsive genes [25]. Furthermore, CDPKs are functionally connected to Ca^2+^/CaM signaling and can influence CAMTA3 activity by modulating Ca^2+^ dynamics [26]. These observations raise the possibility of coordinated regulation between CDPKs and CAMTA3 during cold stress.

In this study, we identify VpCDPK13 as a positive regulator of grapevine cold tolerance. Functional analyses revealed that VpCDPK13 enhances cold resistance by mitigating ROS accumulation and maintaining membrane integrity. Importantly, we discovered that VpCDPK13 interacts with and phosphorylates VpCAMTA3 at multiple serine residues, and thereby enhancing VpCAMTA3-mediated cold tolerance in grapevine. These results reveal a phosphorylation-dependent regulatory module: phosphorylation of VpCAMTA3 by VpCDPK13 enhances plant cold tolerance.

## Results

### Cold-activated VpCDPK13 is an evolutionarily conserved protein kinase

In a previous study, we found that VpCDPK13 positively regulates powdery mildew resistance in grapevine [27]. Given that CDPKs frequently participate in multiple stress-response pathways, we investigated whether VpCDPK13 also contributes to cold tolerance. Transcript analysis revealed that *VpCDPK13* expression in leaves was rapidly induced within 1–4 h at 4°C, followed by a decline, whereas its expression in roots gradually increased after 8 h of cold treatment. In contrast, transcript levels in stems showed no significant change (Supplementary Fig. S1A). Subcellular localization analysis showed that VpCDPK13 was distributed both in the nucleus and the endoplasmic reticulum (ER) (Supplementary Fig. S1B). Notably, after exposure to 4°C, the green fluorescent protein (GFP) signal in the nucleus was markedly enhanced, indicating increased nuclear accumulation of VpCDPK13 under cold stress. According to the phylogenetic analysis as well as comparison of sequences alignments, we observed that VpCDPK13 has conserved domain involved in kinase function and calcium ion binding, with high similarity to each other and closely related to VvCDPK13, indicating strong evolutionary conservation within the CDPK13 subgroup (Supplementary Fig. S2A and B). Overall, these results demonstrate that VpCDPK13 encodes a conserved, cold-inducible Ca^2+^-dependent protein kinase that may function as an early signal transducer in cold-stress responses.

### VpCDPK13 positively regulates cold tolerance in grapevine

**Figure 1:**
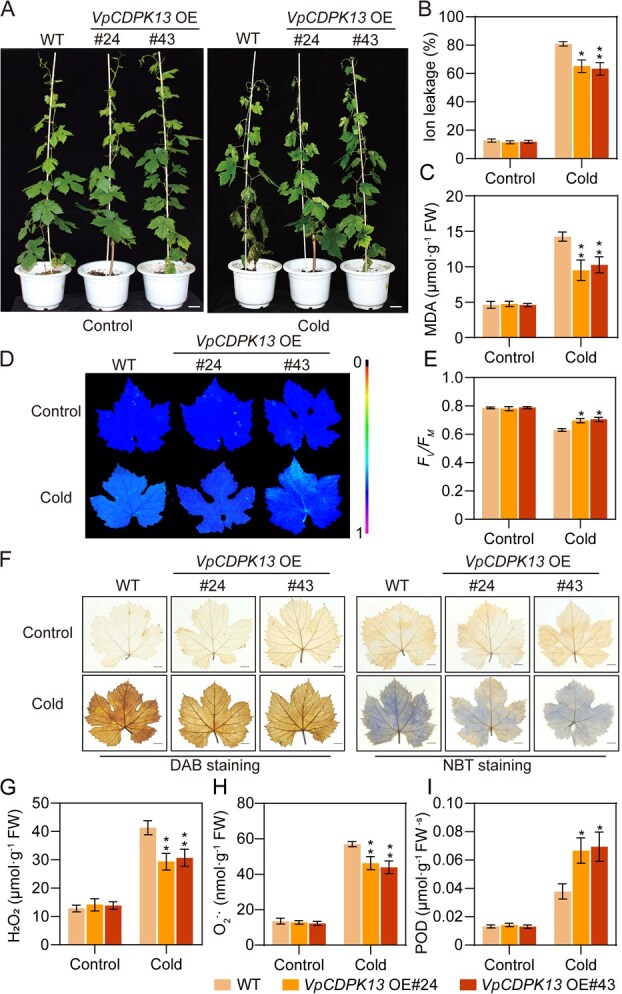
Overexpression of *VpCDPK13* enhances cold tolerance in grapevine. (A) Representative phenotypes of WT and *VpCDPK13* overexpression (OE) grapevine lines (#24 and #43) at control (22°C) and cold (−2°C). Scale bars, 10 cm. (B) Ion leakage of WT and OE lines after exposure to different freezing temperatures at control (0°C) and cold (−6°C). The analysis of MDA content (C), chlorophyll fluorescence (D and E), DAB and NBT staining (F), H₂O₂ content (G), O₂^−^· content (H), and POD activity (I) in WT and OE plants at control (22°C) and cold (−2°C) conditions. The false color scale in (D) represents values ranging from 0 to 1, representing leaf damage levels. Scale bars in (F) are 1 cm. Error bars indicate SD (*n* = 3 in A, *n* = 10 in B), *n* = 5 in (C, E, G, H, and I). Significant differences were determined using Student’s *t*-test (^*^P < 0.05; ^**^P < 0.01; ^***^P < 0.001).

**Figure 2:**
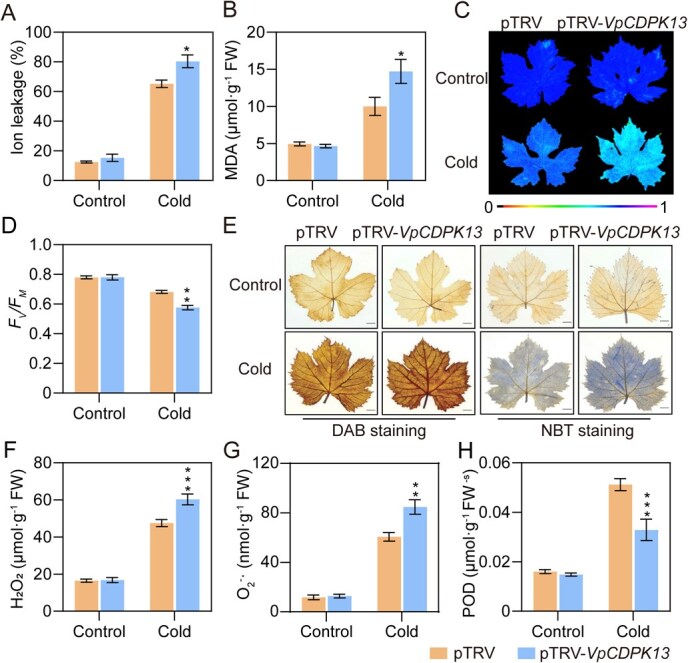
Silencing of *VpCDPK13* reduces cold tolerance in grapevine. (A) Electrolyte leakage assays of pTRV plants and *VpCDPK13*-silenced lines following exposure to freezing temperatures at control (0°C) and cold (−6°C). The analysis of MDA content (B), chlorophyll fluorescence (C and D), DAB and NBT staining (E), H₂O₂ content (F), O₂^−^· content (G), and POD activity (H) in pTRV and *VpCDPK13*-silenced plants at control (22°C) and cold (−2°C) conditions. The false color code in (C) ranges from 0 to 1, representing leaf damage levels. Scale bars in (E) are 1 cm. pTRV, empty VIGS vector; pTRV-*VpCDPK13*, knockdown construct for *VpCDPK13*. Error bars indicate SD (*n* = 10 in A), *n* = 5 in (B, D, F, G, and H). Significant differences were determined using Student’s *t*-test (^*^P < 0.05; ^**^P < 0.01; ^***^P < 0.001).

To investigate the role of VpCDPK13 in cold-stress responses, we generated transgenic overexpression (OE) and virus-induced gene silencing (VIGS) plants. Molecular analyses confirmed stable overexpression of VpCDPK13 in two independent OE lines (Supplementary Fig. S3A). VpCDPK13 OE plants exhibited elevated transcript levels of *VpCDPK13* compared with wild type (WT) (Supplementary Fig. S3B). After cold treatment, *VpCDPK13* OE plants maintained greener leaves and reduced wilting than WT (Fig. 1A). Consistently, *VpCDPK13* OE lines displayed significantly lower electrolyte leakage and malondialdehyde (MDA) under cold conditions (Fig. 1B and C) and maintained higher maximum quantum efficiency of PSII (*F_v_/F_m_*) (Fig. 1D and E). Histochemical staining showed weaker 3,3′-diaminobenzidine (DAB) and nitro blue tetrazolium (NBT) accumulation in *VpCDPK13* OE leaves than in WT, indicating reduced accumulation of hydrogen peroxide (H_2_O_2_) and superoxide (O_2_^−^·) (Fig. 1F). Consistently, *VpCDPK13* OE plants displayed lower ROS levels and higher peroxidase (POD) activity than WT under cold stress (Fig. 1G–I). Conversely, VIGS-based suppression of *VpCDPK13* reduced its transcript abundance and compromised cold tolerance, compared with the empty TRV control (Supplementary Fig. S5A). Silenced plants exhibited elevated electrolyte leakage and higher MDA content (Fig. 2A and B) and reduced *F_v_/F_m_* compared with pTRV plants under cold stress (Fig. 2C and D). In addition, silenced plants showed increased ROS accumulation (Fig. 2E–G) and decreased POD activity under cold stress (Fig. 2H). Taken together, these results demonstrate that VpCDPK13 acts as a positive regulator of cold tolerance in grapevine by enhancing antioxidant capacity and maintaining photosynthetic efficiency.

### VpCDPK13 interacts with the cold-positive regulator VpCAMTA3

**Figure 3:**
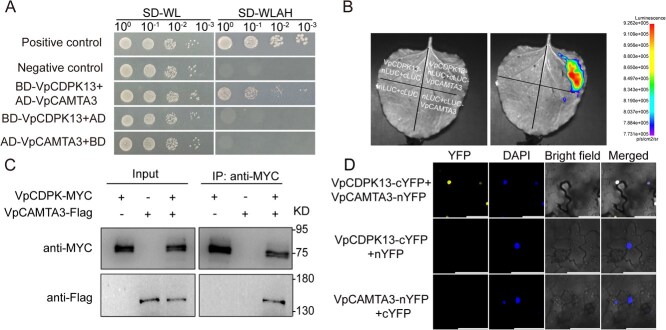
VpCDPK13 and cold positive regulator VpCAMTA3 interaction in grapevine. (A) Y2H experiment shows that VpCDPK13 interacts with VpCAMTA3. SD-L/W and SD-L/W/H/A denote selective media lacking Leu/Trp or Leu/Trp/His/Ade. (B) Split-LUC verifies the interaction between VpCDPK13 and VpCAMTA3 in tobacco. (C) Co-IP confirms the interaction between VpCDPK13 and VpCAMTA3 in tobacco. Immunoprecipitation is carried out using anti-MYC antibody, while for immunoblot, both anti-MYC and anti-Flag are used. (D) Interaction between VpCDPK13 and VpCAMTA3 by BiFC assay. The YFP C-terminal fragment is fused with VpCDPK13, while the N-terminal fragment is conjugated with VpCAMTA3. Nuclear localization is confirmed by DAPI staining. Scale bar, 50 μm.

To explore the molecular mechanisms of VpCDPK13, we performed yeast two-hybrid (Y2H) screening identified VpCAMTA3 as a candidate interactor. Direct interaction between VpCDPK13 and VpCAMTA3 was confirmed by Y2H experiments (Fig. 3A). This interaction was further validated by split luciferase complementation (Split-LUC) in tobacco (*Nicotiana benthamiana*), which produced in leaves coexpressing VpCDPK13-nLUC and cLUC-VpCAMTA3 (Fig. 3B). Co-immunoprecipitation (Co-IP) analysis also confirmed their physical association, as VpCAMTA3-Flag was coprecipitated with VpCDPK13-MYC (Fig. 3C). Moreover, bimolecular fluorescence complementation (BiFC) assays revealed reconstituted YFP fluorescence in cells coexpressing VpCDPK13-cYFP and VpCAMTA3-nYFP, providing additional *in vivo* evidence of their interaction (Fig. 3D), providing strong evidence for their physical association *in vivo*.

**Figure 4:**
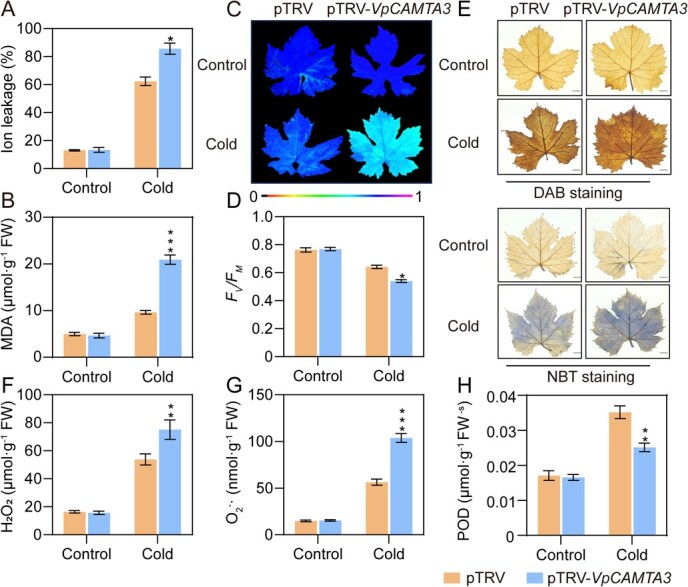
Silencing of *VpCAMTA3* reduces cold tolerance in grapevine. (A) Electrolyte leakage in pTRV plants and *VpCAMTA3*-silenced plants exposed to freezing temperatures at control (0°C) and cold (−6°C). The analysis of MDA content (B), chlorophyll fluorescence (C and D), DAB and NBT staining (E), H₂O₂ content (F), O₂^−^· content (G), and POD activity (H) in pTRV and *VpCAMTA3*-silenced plants at control (22°C) and cold (−2°C) conditions. The false color code in (C) ranges from 0 to 1, representing leaf damage levels. Scale bars in (E) are 1 cm. Error bars indicate SD (*n* = 10 in A), *n* = 5 in (B, D, F, G, and H). Significant differences were determined using Student’s *t*-test (^*^P < 0.05; ^**^P < 0.01; ^***^P < 0.001).

In addition, we investigated whether VpCAMTA3 plays a role in cold tolerance. Expression analysis showed that *VpCAMTA3* was rapidly induced in leaves and roots by cold stress, whereas expression in stems remained largely unchanged (Supplementary Fig. S4A). Subcellular localization showed that VpCAMTA3 was localized in the nucleus (Supplementary Fig. S4B). VIGS-mediated silencing of *VpCAMTA3* significantly reduced its transcript abundance (Supplementary Fig. S5B). pTRV-*VpCAMTA3* plants exhibited increased electrolyte leakage (Fig. 4A), elevated MDA levels (Fig. 4B), reduced *Fv/Fm* (Fig. 4C and D), enhanced ROS accumulation (Fig. 4E–G), and decreased POD activity (Fig. 4H) compared with pTRV plants under cold stress (Fig. 4). These findings indicate that VpCAMTA3 acts as a positive regulator of grapevine cold tolerance.

### VpCDPK13 phosphorylates VpCAMTA3 at multiple sites

**Figure 5:**
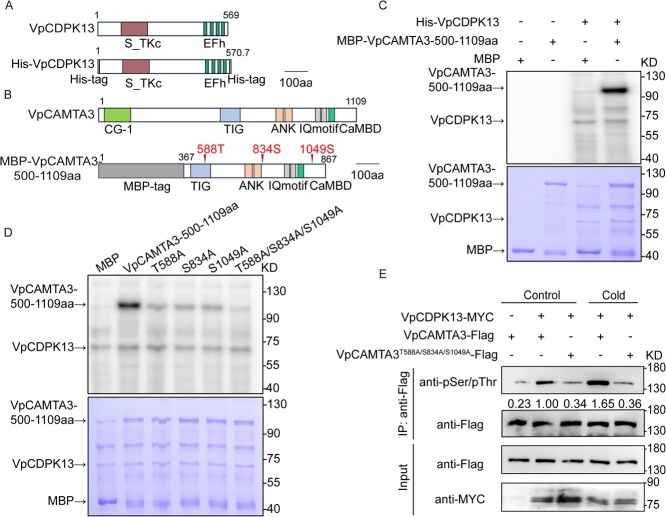
VpCDPK13 phosphorylates VpCAMTA3 *in vitro* and *in vivo*. **(A)** Schematic diagram of the VpCDPK13 protein sequence. His-VpCDPK13, VpCDPK13 fused with two His tags. (B) Schematic diagram of the VpCAMTA3 protein sequence showing three putative phosphorylation sites (T588, S834, and S1049). MBP-VpCAMTA3–500-1109 aa, VpCAMTA3–500-1109 aa fused with an MBP tag; (C) *In vitro* kinase assay showing that His-VpCDPK13 phosphorylates the MBP-VpCAMTA3–500-1109 aa fragment. Phosphorylated proteins were visualized by autoradiography, and CBB staining verified equal loading. (D) *In vitro* phosphorylation analysis of single and triple alanine mutants of VpCAMTA3. Phosphorylation was greatly diminished in the triple mutant (T588A/S834A/S1049A), indicating that these residues are critical for phosphorylation. CBB staining shows protein loading. (E) *In vivo* phosphorylation assay showed that VpCDPK13 phosphorylates VpCAMTA3 in *N. benthamiana* leaves. Control (22°C) and cold (4°C) treatment were applied to explore the phosphorylation of VpCAMTA3 by VpCDPK13 under cold stress.

Given the physical interaction between VpCDPK13 and VpCAMTA3, we investigated whether VpCAMTA3 serves as a phosphorylation substrate of VpCDPK13. Full-length VpCDPK13 protein was successfully expressed in *Escherichia coli* (Fig. 5A). Owing to difficulties in obtaining soluble full-length VpCAMTA3 protein, the C-terminal fragment of VpCAMTA3 (amino acids 500–1109) was purified for subsequent analyses (Fig. 5B). *In vitro* kinase assay demonstrated that VpCDPK13 phosphorylated VpCAMTA3 (500–1109 aa) (Fig. 5C). To identify specific phosphorylation sites, liquid chromatography–tandem mass spectrometry (LC–MS/MS) analysis was performed, which revealed three candidate phosphorylation residues within this region: Thr588, Ser834, and Ser1049 (Supplementary Fig. S6). To evaluate the contribution of these residues to VpCAMTA3 phosphorylation, single and triple alanine substitution mutants were generated. Substitution of Thr588, Ser834, or Ser1049 individually resulted in a noticeable reduction in phosphorylation compared with the WT protein, whereas the triple mutant (T588A/S834A/S1049A) exhibited a pronounced decrease in phosphorylation (Fig. 5D). Then, to determine whether VpCDPK13 phosphorylates VpCAMTA3 *in vivo*, we coexpressed *VpCDPK13-MYC* and *VpCAMTA3-Flag* in *N. benthamiana* leaves and performed an immunoblotting analysis *in planta* using an anti-pSer/pThr antibody to detect phosphorylated S and T residues on VpCAMTA3. The results showed that, under control conditions, VpCDPK13 can phosphorylate VpCAMTA3 *in vivo*. Phospho-dead mutants VpCAMTA3^T588A/S834A/S1049A^ significantly decreased the phosphorylation level of VpCAMTA3, consistent with *in vitro* results (Fig. 5D). These results also suggest that additional phosphorylation sites may be present. Compared with the control condition, cold stress further enhanced VpCDPK13-mediated *in vivo* phosphorylation of VpCAMTA3. Phospho-dead mutants VpCAMTA3^T588A/S834A/S1049A^ decreased the phosphorylation level to one-fifth of the WT under cold (Fig. 5E). These observations indicate that VpCDPK13 phosphorylates VpCAMTA3 at multiple residues within its C-terminal regulatory region, with Thr588, Ser834, and Ser1049 representing the major phosphorylation sites.

### Phosphorylation of VpCAMTA3 enhances cold tolerance

**Figure 6:**
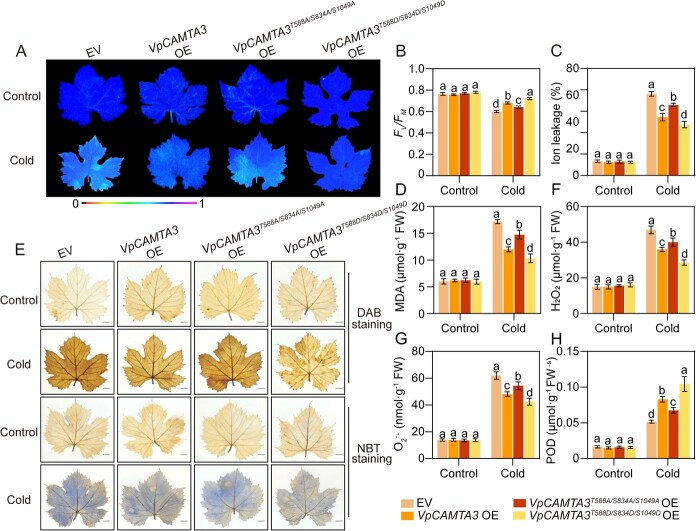
VpCAMTA3 phosphorylation enhances cold tolerance of grapevine. (A and B) Chlorophyll fluorescence imaging and *F_V_/F_m_* quantification of EV, *VpCAMTA3* OE, phospho-dead mutant (*VpCAMTA3^T588A/S834A/S1049A^* OE), and phospho-mimic mutant (*VpCAMTA3^T588D/S834D/S1049D^* OE) at control (22°C) and cold (−2°C). The color scale in (A) indicates the extent of cold-induced damage, from 0 to 1. (C) Electrolyte leakage following freezing treatments at control (0°C) and cold (−6°C). The analysis of MDA content (D), DAB and NBT staining (E), H₂O₂ content (F), O₂^−^· content (G), and POD activity (H) at control (22°C) and cold (−2°C) conditions. Scale bars in (E) are 1 cm. EV, empty overexpression vector. Error bars indicate SD (*n* = 10 in C), *n* = 5 in (B, D, F, G, and H). One-Way ANOVA is performed and followed by Tukey’s multiple comparison test, where different letters show *P* < 0.05.

To explore the biological roles of VpCAMTA3 phosphorylation during cold stress, grapevine leaves with transient overexpression of WT *VpCAMTA3* (*VpCAMTA3* OE), a phospho-dead mutant (*VpCAMTA3^T588A/S834A/S1049A^* OE), and a phospho-mimic mutant (*VpCAMTA3^T588D/S834D/S1049D^* OE) were generated (Supplementary Fig. S5C and D). Chlorophyll fluorescence imaging showed that the *F_v_/F_m_* values of phospho-dead plants and empty vector plants markedly decreased after cold treatment, whereas *Fv/Fm* values remained significantly higher in VpCAMTA3 OE plants and were highest in phospho-mimic plants (Fig. 6A and B). These results indicating that phosphorylation of VpCAMTA3 enhanced the stability of photosystem II under cold.

Consistent with the chlorophyll fluorescence data, phospho-mimic plants exhibited significantly lower ion leakage and MDA content compared to *VpCAMTA3* OE plants, whereas phospho-dead plants showed increased ion leakage and MDA accumulation (Fig. 6C and D), this result suggests that phosphorylation of VpCAMTA3 contributes to the maintenance of membrane integrity under cold conditions. Furthermore, histochemical staining and biochemical analyses supported the enhanced cold tolerance conferred by VpCAMTA3 phosphorylation. Compared with *VpCAMTA3* OE plants, phospho-mimic plants accumulated lower levels of ROS, as indicated by weaker DAB and NBT staining, and displayed higher POD activity. In contrast, phospho-dead plants exhibited greater cold sensitivity, characterized by enhanced ROS accumulation and reduced POD activity (Fig. 6E–H). In summary, these results demonstrate that phosphorylation of VpCAMTA3 is critical for its positive role in cold tolerance.

## Discussion

CDPKs act as pivotal sensors and transducers that decode transient cytosolic Ca^2+^ into specific phosphorylation events, thereby regulating diverse physiological processes, including plant responses to abiotic stress [15, 28, 29]. In this study, we demonstrate that VpCDPK13 acts as a positive regulator of cold tolerance in grapevine, providing mechanistic insight into Ca^2+^-mediated cold-stress signaling in this perennial fruit crop. *VpCDPK13* transcription was rapidly induced by low temperature, and the protein localized to both the nucleus and the ER, with enhanced nuclear accumulation under cold stress (Supplementary Fig. S1). This dynamic localization pattern is consistent with a role for VpCDPK13 in sensing early cold-induced Ca^2+^ signals and relaying them to downstream nuclear targets [13, 14]. Functional analyses revealed that *VpCDPK13* overexpression significantly alleviated cold-induced cellular damage, as evidenced by reduced electrolyte leakage and lipid peroxidation, lower ROS accumulation, and maintenance of higher *Fv/Fm* (Figs 1 and 2). Conversely, transient silencing of *VpCDPK13* compromised these protective responses (Fig. 2). These physiological changes are hallmarks of enhanced cold tolerance and are consistent with previous studies demonstrating that CDPKs contribute to cold adaptation by modulating antioxidant capacity, membrane stability, and photosynthetic performance [17, 20, 30, 31].

CAMTA transcription factors are well-established regulators of cold-responsive gene expression, acting downstream of Ca^2+^/calmodulin signaling [23, 32, 33]. In grapevine, VvCAMTA3 has been implicated in low-temperature responses and antioxidant regulation [24]. Moreover, the ability of VpCAMTA3 to suppress ROS accumulation resembles the mechanism of EjCDPK25 in loquat, which enhances freezing tolerance through modulation of ROS metabolism [19]. In this study, we show that VpCAMTA3 positively regulates cold tolerance, as its silencing led to increased ROS accumulation, impaired membrane integrity, and reduced photosynthetic efficiency under cold stress (Fig. 4). These observations collectively suggest a conserved ‘CDPK-CAMTA-ROS regulatory module’ across plant species.

In this study, we identified that VpCAMTA3 as a direct phosphorylation substrate of VpCDPK13. Phosphorylation assays demonstrated that VpCDPK13 phosphorylates VpCAMTA3 at three C-terminal residues (Thr588, Ser834, Ser1049) *in vitro* and *in vivo* (Fig. 5). Moreover, functional analyses using phospho-dead and phospho-mimic mutants demonstrated that phosphorylation of VpCAMTA3 is required for optimal cold tolerance, as phospho-mimic plants exhibited enhanced photosystem II stability, reduced membrane damage, and improved ROS-scavenging capacity under cold stress (Fig. 6). These results confirm that phosphorylation acts as a positive regulatory switch to potentiate VpCAMTA3-mediated cold response. Notably, this regulatory mechanism differs from the reported regulation of CAMTA3 in *Arabidopsis*, where phosphorylation by MPK3/6 promotes CAMTA3 destabilization [25]. In contrast, VpCDPK13-mediated phosphorylation enhances VpCAMTA3 activity, highlighting species-specific diversification of CAMTA3 regulation and suggesting that distinct kinase modules have evolved to fine-tune cold signaling in different plant lineages. Such divergence may reflect adaptive strategies associated with perennial growth habits and environmental niches of grapevine.

CDPKs decode Ca^2+^ signatures into phosphorylation outputs through context-dependent substrate recognition that involves not only linear motifs (R/K-X-X-S/T; L-X-R-X-X-S/T) but also substrate conformation, proximal modifications, kinase autophosphorylation and cofactors (14-3-3 s) [15, 29, 34, 35]. Although Thr588, Ser834, and Ser1049 represent the major phosphorylation sites identified in this study, residual phosphorylation detected in the triple mutant suggests the presence of additional minor sites. Indeed, potential CDPK phosphorylation motifs of RLHS and KLHS were identified in the N-terminal region of VpCAMTA3, which may contribute to fine regulation of its activity. Further studies will be required to clarify whether these sites are phosphorylated *in vivo* and how they integrate with the VpCDPK13-VpCAMTA3 signaling pathway.

Collectively, our findings support a model in which cold-induced activation of VpCDPK13, which in turn phosphorylates VpCAMTA3 to enhance its regulatory activity, leading to improved ROS homeostasis, membrane stability, and photosynthetic performance under cold stress. This VpCDPK13-VpCAMTA3 module represents a previously uncharacterized regulatory pathway that contributes to grapevine cold tolerance.

## Conclusion

This study identifies VpCDPK13 as a cold-inducible calcium-dependent protein kinase that positively regulates cold tolerance in grapevine. Mechanistically, VpCDPK13 directly interacts with and phosphorylates VpCAMTA3 at Thr588, Ser834, and Ser1049 within its C-terminal region, Phosphorylation of VpCAMTA3 enhances its ability to confer cold tolerance by maintaining photosystem II stability, preserving membrane integrity, and promoting efficient ROS scavenging. The VpCDPK13–VpCAMTA3 signaling module uncovered here provides new insight into cold-stress signaling in grapevine and offers potential molecular targets for improving cold tolerance in perennial fruit crops.

## Materials and methods

### Plant materials and growth conditions

*Vitis pseudoreticulata* ‘Baihe-35-1’ was used as the plant material for RNA extraction, tissue culture, and transgenic experiment according to standard procedures [27]. Cuttings were used for vegetative multiplication and the plants cultivated in pots containing a mix of peat, vermiculite, and perlite at the volumetric proportion (4:1:1), respectively. Plants were grown under greenhouse conditions (day length = 16 h). Temperature (22°C–25°C), relative humidity (70%–80%) and a light intensity of (200 μmol m^−2^ s^−1^) were regulated throughout the experiment t; regular irrigation was applied in conjunction with a Hoagland nutrient solution when necessary.

### Bioinformatics analysis

BLAST searches were performed against the GenBank database to retrieve the homologous protein sequences of VpCDPK13. Multiple sequence alignments were generated using the online tools ClustalW and ESPript 3.0 (https://espript.ibcp.fr; accessed on 10 October 2025), and phylogenetic trees were established with MEGA 11 employing the neighbor-joining algorithm.

### RNA extraction and qRT-PCR

Grapevine RNA was isolated tissues using the CTAB protocol. cDNA was synthesized using the RevertAid kit (Thermo), and quantitative reverse transcription PCR (qRT-PCR) was performed on a CFX96 system (Bio-Rad, USA). Relative expression was calculated by the 2^-ΔΔCt^ method using Actin as an internal control [36].

### Subcellular localization

To investigate the intracellular distribution of *VpCDPK13* and *VpCAMTA3*, their full-length coding sequences were individually fused to the GFP reporter in the pCAMBIA2300-GFP vector. The recombinant constructs were introduced into *Agrobacterium* strain GV3101 and transiently expressed in leaves of tobacco. An ER marker, WAK2-mCherry-HDEL, was constructed by linking the signal peptide of AtWAK2 to the N-terminus and the ER retention motif HDEL to the C-terminus [37]. After 72 h culture at 22°C, the plants were inoculated with bacteria and imaged on a Leica TCS SP8 confocal microscope (Germany).

### Transient transformation of grapevine leaves

To examine transient genetic manipulation in grapevine leaves, full-length cDNA of *VpCAMTA3* was cloned into pCAMBIA2300-GFP vector to perform transient expression experiments. Meanwhile, the part of cDNA fragments of *VpCDPK13* or *VpCAMTA3* was cloned into the vector pTRV for RNA interference test. The constructed vectors, along with their respective control vector (pCAMBIA2300-GFP and pTRV) into GV3101 bacteria. The resulting agro-infiltrated *A. tumefaciens* suspensions were used to infiltrate grapevine leaves by vacuum infiltration, each treatment had at least 20 leaves. Phenotypic observations were conducted after 3 days of incubation under ambient temperature conditions. Additionally, the expression levels of related genes in leaves were analyzed by qRT-PCR, and the levels of related proteins were detected by western blotting.

### Cold treatments and determination of ion leakage

Two-month-old grape tissue culture seedlings (*V. pseudoreticulata*) were exposed to 4°C for 0, 1, 4, 8, 12, and 24 h. Plant tissues were separately collected at each time point for qRT-PCR analysis to examine the cold-induced expression of *VpCAMTA3* and *VpCDPK13*. For the low-temperature treatment experiment, 3-month-old *VpCDPK13* OE and WT were exposed to −2°C for 6 h to observe phenotypic responses.

Ion leakage was detected as described [38–40].

### Chlorophyll fluorescence imaging

The maximum quantum efficiency of PSII (*F_v_/F_m_*) was detected as described [41]. Plants were subjected at −2°C for 2 h. The *F_v_/F_m_* values were subsequently determined using an IMAGING-PAM chlorophyll fluorometer (Germany).

### ROS quantification and histochemical detection under cold stress

ROS accumulation, including H_2_O_2_ and O_2_^−^·, was analyzed following cold-stress treatment. Leaf samples were collected immediately for quantitative determination and histochemical staining.

For the quantification analysis, plants were ground in liquid N and analyzed by commercial reagents kits from Comin Biotechnology, Determination of H₂O₂ and O_2_^−^· Quantification of H₂O₂ and O_2_^−^· were performed using a commercial kit (China), following instructions provided by the supplier. Histochemical detection of H₂O₂ was performed by soaking leaves into DAB dye (1 mg/ml). Leaves were incubated in the dark at 25°C (~8–10 h after treatment). Afterward, chlorophylls were extracted by a destaining solution of ethanol-distilled water-acetic acid-glycerol (vol/vol = 8:1:1:1), which allowed easy viewing of stained regions. Superoxide radical accumulation was determined by NBT, which is stained using NBT at the concentration of 1 mg/ml and kept in the dark for one night. For photographing purposes, the stained specimens were then boiled with a mixture of ethanol, lactic acid, and glycerol (in this order at volumes 3:1:1) in an attempt to remove other colors. All experiments were performed in triplicate for the purpose of statistics verification.

### Determination of MDA content and POD activity

MDA content and POD enzyme activity were measured following standard protocols included in commercial test kits (Comin Biotechnology, China). After the treatment of −2°C (2 h), leaves were immediately snap-frozen in liquid nitrogen and then ground to powder. The samples were analyzed and ~0.1 g of plants was extracted using respective extraction medium provided for kits and centrifuged to collect supernatant. Determination of MDA content by TBA method, where OD is the absorbance values measured at 532, 600, and 450 nm by a spectrophotometer. POD activity: the change in absorbance values (at a wavelength of 470 nm) over time as guaiacol is oxidized by H_2_O_2_.

### Split luciferase complementation assay

To evaluate the interaction between VpCDPK13 and VpCAMTA3 *in planta*, we first constructed the related plasmids. The coding DNA sequence (CDS) of *VpCDPK13* was inserted into the pCAMBIA1300-nLuc vector, whereas the CDS of *VpCAMTA3* was fused to the C-terminal luciferase fragment in pCAMBIA1300-cLuc. Then the constructed vector was transformed into GV3101 strain, and the OD600 of the *Agrobacterium* suspension to be injected was maintained at ~0.6, and then coexpressed in tobacco. Luminescence signals were recorded 3 days postinfiltration using a Tanon 4600 chemiluminescence imaging system.

### Yeast two-hybrid assay

To perform a Y2H screen, we cloned the full-length *VpCDPK13* in pGBKT7 bait plasmid while *VpCAMTA3* was cloned in pGADT7 prey plasmid. The resulting vectors were cotransformed to AH109 strain of yeast. Yeast transformants were initially selected on SD medium minus Leu and Trp, followed by transfer on quadruple dropout plates (without leucine, tryptophan, adenine and histidine), in order to assess protein–protein interaction.

### BiFC assay

For subcellular interaction visualization, *VpCDPK13* and *VpCAMTA3* were fused to the C- and N-terminal halves of YFP, respectively. Recombinant constructs were delivered into GV3101 and transiently expressed in tobacco leaves. Fluorescence signals were detected using confocal laser scanning microscopy.

### Co-IP assay

To confirm the interaction between VpCDPK13 and VpCAMTA3 *in vivo*, their full-length CDSs were cloned into pCAMBIA1305-3MYC and pCAMBIA1305-3Flag vectors, respectively. Recombinant plasmids were transformed into GV3101 and co-infiltrated into 4-week-old tobacco leaves. After a 3-day incubation, total proteins were extracted in buffer containing 150 mM NaCl, 5 mM DTT, 5 mM EDTA, 10% glycerol, 50 mM Tris–HCl (pH 7.5), 1 mM PMSF, 1% NP-40, and protease inhibitors. Protein extracts were incubated overnight at 4°C with rabbit anti-MYC antibody (MA9038; Abmart). Immune complexes were captured using protein A/G agarose beads and washed repeatedly with immunoprecipitation buffer. Precipitated proteins were eluted in SDS loading buffer, denatured, and analyzed by immunoblotting with anti-MYC and anti-Flag antibodies.

### *In vitro* phosphorylation assay

The CDS of *VpCDPK13* was subcloned into pET-28a (+), while the C-terminal region of VpCAMTA3, including both WT and site-directed mutant forms (T588A/S834A/S1049A), was inserted into pMAL-c5X. Recombinant proteins were expressed in Rosetta-gami (DE3) and purified using Ni-NTA agarose or amylose affinity chromatography. Purified kinase and substrate proteins were incubated at 30°C for 30 min in kinase buffer (0.5 mM CaCl_2_, 20 mM Tris–HCl, 1 mM DTT, 2.5 mM MnCl_2_, and 2 μCi [γ-^32^P] ATP). Reactions were terminated with SDS loading buffer followed by heat denaturation. Proteins were resolved by 8% SDS-PAGE and stained with Coomassie Brilliant Blue (CBB) to verify equal loading. Radioactive phosphorylation signals were detected by autoradiography.

### *In vivo* phosphorylation assay

Full-length *VpCDPK13* and *VpCAMTA3* sequences were cloned into MYC- and Flag-tagged vectors, respectively, and transiently coexpressed in tobacco leaves through GV3101-mediated infiltration. The fusion proteins, VpCAMTA3-Flag, were immunoprecipitated using anti-Flag antibody-coated magnetic beads. Phosphorylation status was determined by immunoblotting with a phospho-Ser/Thr-specific antibody (PM3801ECM, USA). In addition, a phospho-dead mutant construct, VpCAMTA3^T588A/S834A/S1049A^-Flag, was generated and coexpressed with VpCDPK13-MYC to compare phosphorylation levels *in planta*.

### LC–MS/MS analysis

LC–MS was detected as described [42]. Phosphorylated protein samples were separated by 8% SDS-PAGE and visualized by CBB staining. Target bands were excised, destained, reduced with TCEP, and alkylated with CAA in NH₄HCO₃ buffer. After removal of reagents, in-gel digestion was performed overnight using trypsin. Peptides were desalted via C18 columns prior to mass spectrometric analysis. Samples were analyzed on an Orbitrap Fusion Lemos (Thermo). Data processing was conducted using Proteome Discoverer 2.5 software.

### Statistical analysis

Statistical analyses were performed using the SPSS version 26.0 (SPSS, Chicago, IL, USA). Statistically significant differences are indicated by asterisks (^*^P < 0.05; ^**^P < 0.01; ^***^P < 0.001). One-way ANOVA with Tukey’s test was used to test for differences between lines of plants (*P* < 0.05). All values are presented as means ± SD.

## Supplementary Material

Web_Material_uhag121

## Data Availability

All the relevant data supporting the findings of this study are available in the paper and supplementary data. Sequence data of this study can be found in the GenBank (http://www.ncbi.nlm.nih.gov) under the accession numbers: VpCDPK13 (MW167771).
